# Moving Beyond the Phenotypic Correlation Between Anorexia Nervosa and Autism

**DOI:** 10.1002/eat.24332

**Published:** 2024-11-23

**Authors:** Karl Lundin Remnélius, Sven Bölte

**Affiliations:** ^1^ Department of Women's and Children's Health Karolinska Institutet, Center of Neurodevelopmental Disorders at Karolinska Institutet (KIND) Stockholm Sweden; ^2^ Region Stockholm, Child and Adolescent Psychiatry Stockholm Health Care Services Stockholm Sweden; ^3^ Curtin Autism Research Group Curtin University, Curtin School of Allied Health Perth Australia

**Keywords:** anorexia nervosa, autism, autistic traits, eating problems

## Abstract

The recent systematic review and meta‐analysis by Inal–Kaleli et al. is situated within a growing area of research, investigating the relationship between anorexia nervosa and autistic characteristics. Their synthesis of research within the topic finds support for elevated autistic characteristics and autism in individuals with anorexia nervosa and a small but significant correlation between autistic traits and level of eating disorder symptoms. In this commentary, we discuss the findings of Inal‐Kaleli and colleagues and propose further research to generate insights into the nature of this link. We focus on the potential origins of the observed relationship, specific mechanisms, and manifestation of anorexia nervosa in autistic populations, and the influence of sex and gender on the intersection of eating disorders and autism. By addressing these largely unexplored research avenues, future investigations can go beyond the phenotypic correlations and facilitate the development of prevention and intervention suited for individuals experiencing both disordered eating and elevated autistic traits.

## 1 Introduction

The systematic review and meta‐analysis by Inal‐Kaleli et al. (2025) explores the presence of autistic traits and autism diagnosis in populations with anorexia nervosa (AN). In their review, the researchers found elevated autistic traits in AN compared to control groups, and a notable, albeit small, correlation between autistic traits and eating disorder symptoms. In addition, their meta‐analysis of studies using the Autism Diagnostic Observation Schedule (ADOS) in individuals with AN indicates that as many as 29% of individuals with AN meet the cut‐off for autism spectrum condition. As raised by the authors, understanding the overlap between these two conditions is essential to identify the specific treatment needs of this group, given that individuals with AN and elevated autistic traits may have less favorable treatment responses than individuals without pronounced autistic traits. The review by Inal‐Kaleli introduces this research area by providing convincing evidence on the relationship between AN and autism. While the evidence summarized in the review is in favor of a covariation between the conditions, insights regarding the nature of the relationship are still largely lacking. In this commentary, we discuss research areas that are pivotal to clarify the link between AN and autism.

## 2 Disentangling the Origins Driving the Phenotypic Correlations Between AN and Autism

While Inal‐Kaleli and colleagues report a correlation between AN and autism, they leave space for further discussions on *why* the phenotypic association occurs. As discussed by the authors, a possible explanation of the link between these conditions is that they share overlapping etiological factors. Autism is considered a highly heritable condition, and similarly, research supports a substantial genetic influence on AN (Adams et al. 2024). Still, in contrast to the longstanding suggestions of a familial link, few studies have evaluated potential common genetic and environmental influences on the development of the conditions. If the conditions share common genetic factors, it may be of importance to identify early signs of disordered eating among autistic people. (We use identity–first language, e.g. “autistic individual”, rather than person–first language when discussing autism throughout this commentary, in accordance with the preference of many autistic people). Given that a genetic risk or predisposition does not entail genetic determinism, early prevention efforts could be helpful in preventing the development of AN in autistic individuals. To shed further light on the nature of the link, family‐based designs including twin studies, for example, using cross‐twin cross‐trait analyses, can provide early steps toward disentangling potential common genetic and environmental influences from tentatively causal relationships between autism and AN.

Alternatively, the correlations reported in the systematic review may be due to a causal link where one of the conditions contributes to the other. It has been suggested that the effects of starvation increase autistic traits, implying that restrictive eating may precede and cause autistic traits. However, this may not be a sufficient explanation, given that autistic characteristics can remain even after weight restoration, as Inal‐Kaleli et al. (2025) discussed. Conversely, it may also be that autistic traits that are present early in life contribute to the development of AN. For instance, the review reports that individuals with AN show elevated scores compared to control groups on four subscales of the Autism Quotient (AQ), suggesting that cognitive inflexibility along with challenges in social communication may promote restrictive eating in people with elevated autistic traits. Lived experience accounts from autistic women diagnosed with AN corroborate this, expressing that their autistic characteristics, including inflexibility, sensory sensitivities, and social challenges, contribute to the eating disorder (Kinnaird et al. 2019).

The link may also be mediated through other issues common among autistic people. For example, autistic individuals may often experience challenges in emotion regulation which could increase the risk of restrictive eating as a means to control emotions (for a comprehensive review on this topic, see Adams et al. 2024). Similarly, autistic people often have concurrent eating problems, including selective eating and food refusal, which could play a role in the pathway between autism and eating disorders. Therefore, for autistic individuals, early interventions targeting emotion dysregulation and eating problems may reduce the risk of later eating disorders.

Currently, the scarcity of longitudinal studies limits our understanding in this area. While there is some support for links between autistic traits in childhood and eating disorder symptoms in adolescence, the few currently available studies have not consistently supported the link. Future prospective studies could help clarify the relationship and disentangle which specific autistic characteristics are linked to particular eating behaviors later in life. Longitudinal investigations can also give relevant information regarding prevention of the development of AN symptoms. For example, by exploring how timing of autism diagnosis and access to support may moderate the link between autistic traits and AN. If autistic characteristics such as challenges in social communication, atypical sensory processing, or co‐occurring eating problems are involved in the development of AN, early support that facilitates functioning in these areas may reduce the risk of AN.

## 3 Mechanisms and Manifestation of Anorexia Nervosa in Autistic Populations

In order to provide effective treatments for individuals presenting with both AN and autism there is a need for further insights into its manifestation in this group. As discussed above, autistic women perceive that their eating behaviors are tightly intertwined with their autistic characteristics, and that autism‐related challenges are driving forces underlying their restrictive eating, rather than more traditional concerns of body shape and gaining weight (Kinnaird et al. 2019). This suggests that, even if eating behaviors are similar to non‐autistic individuals with AN, the diagnosis may not be as accurate in capturing disordered eating in the autistic population. A broader understanding of disordered eating in autism is thus needed to offer effective support to the group. This requires active assessment of AN and other eating disorders in autistic populations. Here, Avoidant/Restrictive Food Intake Disorder (ARFID) may be particularly relevant given the diagnosis' inclusion of restrictive eating due to sensory sensitivity rather than fear of gaining weight. Since concerns about weight and body shape appear to be less common motivators for restrictive eating among autistic people, it may be that for some autistic individuals with AN, restrictive eating could be better captured by an ARFID diagnosis. Potentially, research including first‐hand accounts of people with lived experience of autism and disordered eating, particularly ARFID, could provide further insights on overlapping aspects and differences across eating disorders.

To gain a broader understanding of eating behaviors among autistic people, future studies should assess eating problems commonly observed in autism alongside classic symptoms of eating disorders. This includes selective eating of a limited range of food items, inflexibility concerning food presentation, and sensory aversion to food textures and smells, along with their impact on everyday functioning and quality of life. Here, factor analysis of items covering autism‐related eating problems and traditional eating disorder symptoms may provide further insights into the overlaps of eating behaviors in autism. Potentially, consistent with other areas of common co‐occurring issues in autism such as anxiety, there may be a need for developing measurements tailored to autism‐specific manifestations of eating disorders.

## 4 The Influence of Sex and Gender on the Link Between AN and Autism

Traditionally, the higher prevalence of autism in males compared to females has guided the inclusion of proportionally more boys and men in autism research. This may result in a male‐bias in our understanding of the condition which could influence how well autism is recognized in females, as noted by the authors of the meta‐analysis. A corresponding predominance of females in the diagnostic prevalence of AN and eating disorder research accordingly contributes to an understanding of the condition that is largely based on the manifestation in females. Considering this, it is not surprising that the samples included in the review were overwhelmingly female (with 19 of the 22 included studies including only female participants). Therefore, it is uncertain if the findings reported in the review generalize to males.

Sex and gender identity may moderate the link between autism and AN. Emerging research support that autistic traits may be more closely connected to issues around eating for females (Lundin Remnélius et al. 2021). Still, autistic males present with largely equivalent challenges in social communication, sensory atypicalities, and cognitive inflexibility, and also face elevated problems concerning eating and mealtimes. Given this context, several mechanisms implicated in AN are present also in autistic males potentially increasing the risk of the eating disorder. However, considering the female predominance in eating disorder populations, there is a surprising dearth of studies exploring the influence of sex and gender on the relationship between autism and eating behavior. Interestingly, one study actively assessing eating disorders in young adults diagnosed with autism and/or ADHD found a more even male‐to‐female ratio compared to the proportions often reported in general population samples (Karjalainen et al. 2016). Potentially, this could be an indication that eating disorders in autistic males may be more common than expected from the prevalence in non‐autistic populations. However, given the meta‐analysis results by Inal‐Kaleli and team, it appears that there is a risk for this group not being recognized or included in research. If males are at risk of having their eating disorder unidentified, this risk may be even more pronounced in autistic males. The general recognition of AN in males may be limited by less‐known male‐typical manifestations and gender bias, which contribute to eating disorders not being as readily attributed to males as they are to females. For autistic males, this may be further exacerbated by autism‐specific AN manifestations, reduced support‐seeking (potentially fueled by AN‐related stigma that may be more pronounced for males), and socio‐communicative challenges in reporting issues. Thus, further research into the potential moderation by sex and gender on the relationship between autism and eating behaviors is warranted. Additionally, qualitative and quantitative methods should be applied to explore experiences of disordered eating and the presence of eating disorder symptoms in autistic males.

## 5 Conclusions

In conclusion, the recent review by Inal‐Kaleli supports that a substantial proportion of individuals with AN also display elevated autistic traits, warranting adapted treatments and services to accommodate the needs in this group. In this commentary, we discuss areas where research is needed to elucidate the nature of this relationship, an essential foundation for developing effective assessment and intervention. We suggest that studies with the following objectives may provide insights into the nature of autism and AN: those aiming to (1) disentangle the causal and etiological factors driving the phenotypic correlations, (2) describe the manifestation of eating disorders in autistic populations, and (3) evaluate the influence of sex and gender on the link.

## Author Contributions

**Karl Lundin Remnélius:** conceptualization, writing – original draft, writing – review and editing. **Sven Bölte:** conceptualization, writing – review and editing.

## Conflicts of Interest

The authors declare no conflicts of interest related to the content of this article. Bölte discloses that he has in the last 3 years acted as an author, consultant, or lecturer for Medice, Roche, and Linus Biotechnology. He receives royalties for textbooks and diagnostic tools from Hogrefe, Ernst Reinhardt, Kohlhammer, and Liber. Bölte is partner in NeuroSupportSolutions International AB.
